# The innate immune cell response to bacterial infection in larval zebrafish is light-regulated

**DOI:** 10.1038/s41598-017-12842-1

**Published:** 2017-10-04

**Authors:** Lucia Y. Du, Hannah Darroch, Pramuk Keerthisinghe, Elina Ashimbayeva, Jonathan W. Astin, Kathryn E. Crosier, Philip S. Crosier, Guy Warman, James Cheeseman, Christopher J. Hall

**Affiliations:** 1 Department of Molecular Medicine and Pathology, Faculty of Medical and Health Sciences, University of Auckland, Auckland, New Zealand; 2 Department of Anaesthesiology, Faculty of Medical and Health Sciences, University of Auckland, Auckland, New Zealand

## Abstract

The circadian clock, which evolved to help organisms harmonize physiological responses to external conditions (such as the light/dark cycle, LD), is emerging as an important regulator of the immune response to infection. Gaining a complete understanding of how the circadian clock influences the immune cell response requires animal models that permit direct observation of these processes within an intact host. Here, we investigated the use of larval zebrafish, a powerful live imaging system, as a new model to study the impact of a fundamental zeitgeber, light, on the innate immune cell response to infection. Larvae infected during the light phase of the LD cycle and in constant light condition (LL) demonstrated enhanced survival and bacterial clearance when compared with larvae infected during the dark phase of the LD cycle and in constant dark condition (DD). This increased survival was associated with elevated expression of the zebrafish orthologues of the mammalian pro-inflammatory cytokine genes, *Tumour necrosis facto*r*-α*, *Interleukin-8* and *Interferon-γ*, and increased neutrophil and macrophage recruitment. This study demonstrates for the first time that the larval zebrafish innate immune response to infection is enhanced during light exposure, suggesting that, similar to mammalian systems, the larval zebrafish response to infection is light-regulated.

## Introduction

Organisms encounter higher risks to infection at certain times of the day. This timing is dependent on environmental conditions associated with increased virulence of pathogens and vulnerability of the host. Given its central role in driving the 24-hour cycle of biological processes to maintain homeostasis, the circadian rhythm is regarded to be critical in regulating the immune system^1–3^. A major environmental driving cue (zeitgeber) of this rhythm is light. In mammals, light entrains the endogenous circadian pacemaker in the suprachiasmatic nucleus (SCN) of the brain, which then synchronizes the circadian clocks of peripheral tissues throughout the body. This involves neuronal signaling and hormones, such as melatonin, serotonin and glucocorticoids^2–6^. At the cellular level, light can induce the expression of the clock genes, period circadian protein (PER) and cryptochrome (CRY), which feedback to repress their own expression by the dimerized transcription factors, brain and muscle aryl hydrocarbon receptor nuclear translocator (ARNT)-like 1 (BMAL1) and circadian locomotor output cycles kaput (CLOCK)^2–4^. This sets up the core transcription-translation feedback loop that generates the circadian rhythm at the molecular level^2–4^. Therefore, the integration of zeitgebers, such as light, and physiological processes is crucial for the optimal functioning of organisms in the face of daily environmental changes.

Circadian regulation is critical in order to mount an efficient immune response without the expense of constant immune activation. Pioneering studies have shown survival variation in response to bacterial challenge in mice, with greater lethality following administration during the transition from the resting to active period^7,8^. Subsequent studies have revealed that circadian rhythms regulate numerous immune functions and parameters, such as host-pathogen interactions, leukocyte recruitment, phagocytosis and cytokine production^2,3,9^. It has been demonstrated that the immune system is better at clearing bacterial infection during the active phase in mice, which coincides with a greater induction of pro-inflammatory cytokines^10^. In part, oscillations in the immune response can be explained by the presence of a molecular clock in hematopoietic-lineage cells, such as macrophages^11^. As a result, current efforts are directed towards investigating the molecular mechanisms underlying the circadian-regulated immune response by studying the role of core clock components. This knowledge will provide the opportunity to develop chronotherapies to treat chronic inflammatory disorders and immune-related dysregulations.

Although some studies have shown the direct antimicrobial effect of light on microbes, it is unclear how it affects the innate host defense response^12,13^. To gain a complete understanding of how light regulates the immune response to infection requires live imaging of these processes with an intact animal model. At present, this is difficult to achieve with conventional models, such as the mouse. The larval zebrafish (*Danio rerio*) is a well-established vertebrate model for studying the circadian and immune systems due to conservation with mammals of clock machinery and immune cell lineages^14–17^. Like mammals, their circadian rhythm is light entrainable and rhythms in the pineal gland’s production of melatonin are clock and light controlled^18^. The genetically tractable nature of the model system facilitates the dissection and analysis of molecular clock components to build upon the fundamental knowledge that have been primarily derived from *Drosophila* and mouse studies^19^. Additionally, the optical transparency of larval zebrafish and availability of transgenic reporter lines possessing fluorescently marked innate immune cells, such as neutrophils and macrophages, provide the unique opportunity to live image these cells and their interactions with microbes within whole larvae, leading to important new insights into host defense mechanisms^20–24^. The innate immune response to infection can also be studied in isolation from the adaptive immune system as larval zebrafish lack a fully functional adaptive immune system prior to four to six weeks post-fertilization^25^. So far, studies using this model have revealed rhythmicity in the phagocytic activity of leukocytes and migration of neutrophils and light-dependent changes in serum immune parameters^26–28^. However, to date, it has not been reported whether the larval zebrafish innate immune cell response to infection is light - dependent.

In this study, we investigated whether the innate immune response to infection in larval zebrafish is influenced by light. We show that the innate immune response to *Salmonella enterica* serovar Typhimurium (hereafter referred to as *Salmonella*) infection was enhanced during light exposure. This is likely to be mediated by the greater expression of the pro-inflammatory cytokine genes, *tumor necrosis factor a* (*tnf-a*), *chemokine (C-X-C motif) ligand 8a* (*cxcl8a*) and *interferon gamma 1-1* (*ifng1-1*), and the elevated recruitment of neutrophils and macrophages to the site of infection, suggesting that the innate immune response in larval zebrafish is light-regulated. The powerful experimental techniques available using this model will serve as a strong platform to uncover new mechanistic insights into how light influences the innate immune cell response during infection.

## Results

### Validation of light-induced rhythm in larval zebrafish

**Figure 1:**
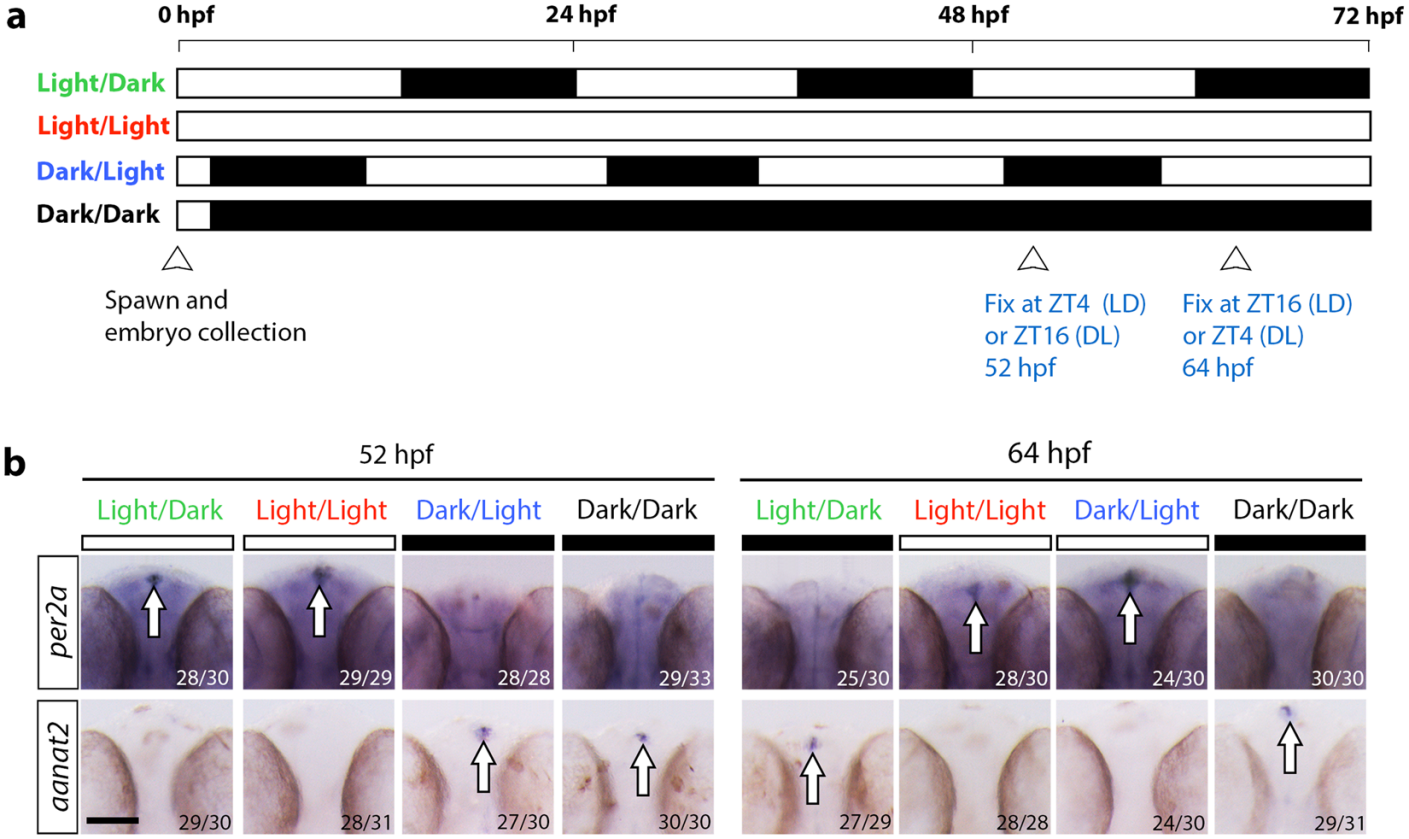
Validation of light-induced rhythmicity by assessing expression of the clock output genes, *per2a* and *aanat2*, under different light conditions. (**a**) Schematic outlining the light conditions and subsequent experimental manipulations. (**b**) Expression analysis of *per2a* and *aanat2* at 52 hpf and 64 hpf, as detected by whole mount *in situ* hybridization (WMISH) for larvae raised under the different light conditions, as shown in (**a**). ZT, zeitgeber time where ZT0 represents start of the light phase; bars above WMISH images denote the light setting at the time of fixation, white bar = light, black bar = dark; numbers represent number of larvae with displayed phenotype; hpf, hours post-fertilization; arrowheads indicate the time of expression analysis; scale bar, 100 μm; arrows mark *per2a* or *aanat2* expression in the pineal gland.

Prior to investigating the immune response to infection under different light conditions, we wanted to confirm if our light treatments influenced the expression of light-responsive genes in 2 days post-fertilization (dpf) larval zebrafish. Light is a strong stimulus for entraining circadian rhythms in developing zebrafish embryos^18,29^. The earliest circadian output signal detected in zebrafish is the rhythmic secretion of melatonin from the pineal gland^30,31^. Production of melatonin involves the key pineal enzyme, *arylalkylamine-N-acetyltransferase* (*aanat2*), which is first detected at 22 hours post-fertilization (hpf) ^31^. The repression of *aanat2* expression by the light-inducible transcriptional regulator, Per2a, restricts melatonin levels to peak during the dark phase^32^. For this reason, we investigated *per2a* and *aanat2* expression by whole mount *in situ* hybridization (WMISH) analysis under the following light conditions: 14-hour light/10-hour dark cycle (LD), constant light (LL), reversed 14-hour light/10-hour dark cycle (DL) and constant darkness (DD) (Fig. 1a). The specific time points, ZT4 at 52 hpf and ZT16 at 64 hpf under the LD cycle, were selected for expression analysis as they corresponded to periods of high *per2a* and *aanat2* expression, respectively^18^. Consistent with a previous study, expression of *per2a* mRNA was detected globally with strong expression in the pineal gland during the light phase at ZT4 (52 hpf) but not during the dark phase at ZT16 (64 hpf) under the LD condition (Fig. 1b)^18^. This was in contrast to *aanat2*, which was only expressed in the pineal gland during the dark phase at ZT16 (64 hpf) (Fig. 1b). By reversing the light condition, embryos exposed to the DL cycle expressed *per2a* and *aanat2* in the pineal gland exclusively during the light (ZT4 at 64 hpf) and dark (ZT16 at 52 hpf) phases, respectively (Fig. 1b). Under constant conditions, only *per2a* was expressed at both time points under LL whereas the opposite was observed for *aanat2* under the DD condition (Fig. 1b).

**Figure 2:**
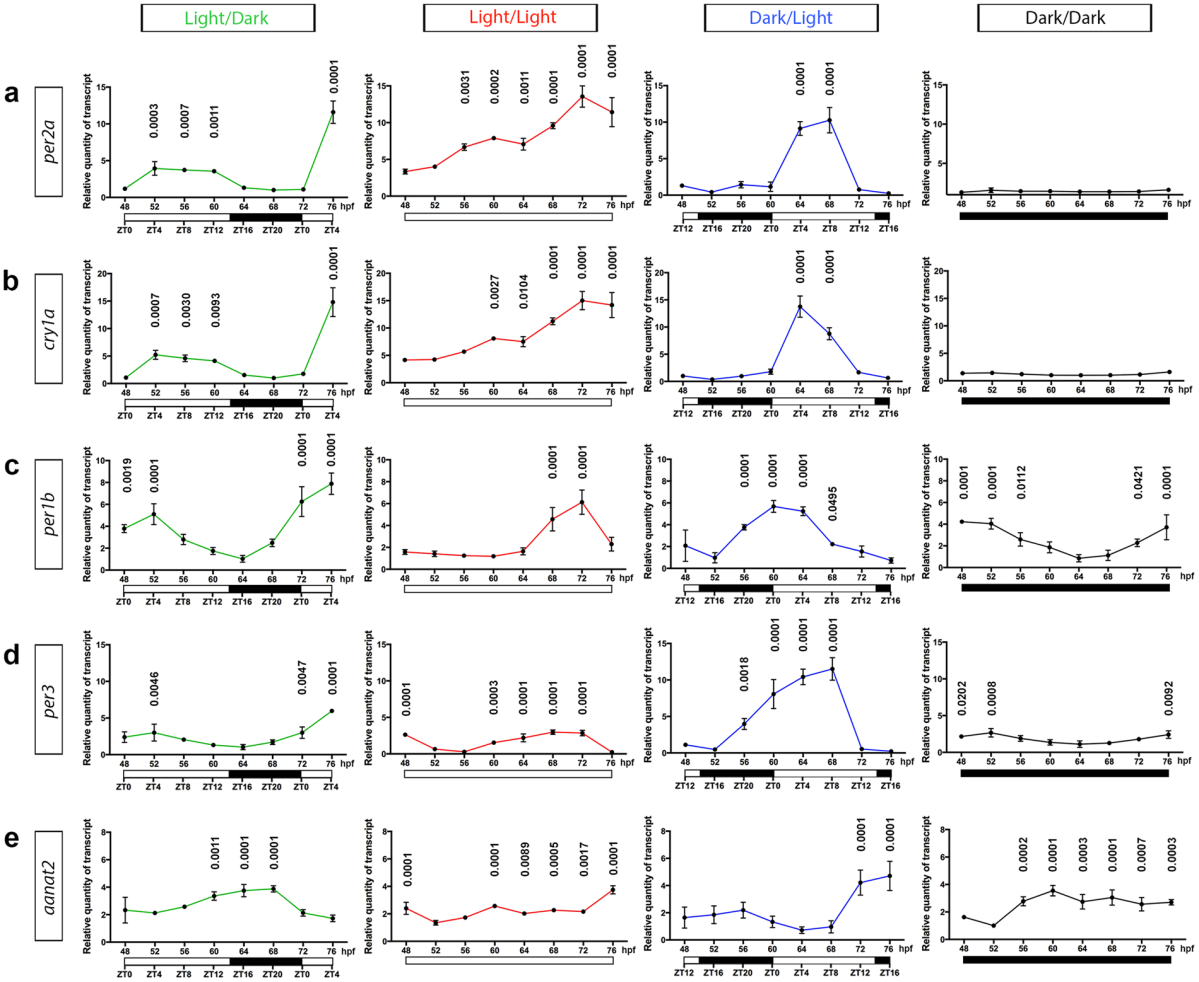
Expression levels of clock genes and *aanat2* display a diel rhythm. (**a**–**d**) qPCR analysis of the clock genes, *per2a, cry1a, per1b* and *per3*, respectively, from whole larvae under light/dark, constant light, dark/light and constant dark conditions from 48 hpf to 76 hpf. (**e**) qPCR expression analysis of *aanat2* from whole larvae under light/dark, constant light, dark/light and constant dark conditions from 48 hpf to 76 hpf. Data represents three biological replicates; data shown as mean ± s.d.; the relative quantity of transcript for each gene, in all conditions, were normalized to the lowest value in the dark/dark group (48, 64, 64, 64 and 52 hpf time points for *per2a*, *cry1a*, *per1b*, *per3*, and *aanat2*, respectively); p-values were calculated by one-way ANOVA with Dunnett’s multiple comparisons test using the lowest data value for each light condition as the reference; hpf, hours post-fertilization; ZT, zeitgeber time where ZT0 represents start of the light phase.

Expression results obtained from WMISH were then validated by analyzing the relative transcript levels of *per2a* and *aanat2*, within whole larvae, over a 28-hour time course from 48 hpf to 76 hpf using qPCR. This analysis was expanded by the inclusion of an additional light-inducible clock gene, *cry1a*, as well as two other clock genes, *per1b* and *per3*, which are known to be synchronized by light^14,33–35^. Similar to *per2a* (Fig. 2a), *cry1a*, *per1b* and *per3* expression levels displayed a pattern, peaking during the light phase under LD and DL conditions (Fig. 2b–d). The diel rhythmicity of these patterns was confirmed by cosinor analysis (Supplementary Fig. 1a–d). The relative transcript levels of *per2a, cry1a﻿, per1b ﻿and﻿ per3* was much higher at 76 hpf (ZT4) compared with 52 hpf (ZT4) under LD conditions (Fig. 2a–d). Both *per2a* and *cry1a* were expressed at lower levels under DD conditions whereas their expression level increased over time under the LL condition in an arrhythmic manner, unlike *per1b* and *per3* (Fig. 2a–d). As expected, expression of *aanat2* in the pineal gland was largely restricted to the dark phase (Fig. 1b) and was rhythmic under LD and DL but arrhythmic under constant conditions (Fig. 2e and Supplementary Fig. 1e). This expression profile was antiphasic to that of *per2a* mRNA in both LD and DL conditions (Fig. 2a,e and Supplementary Fig. 1a,e). When comparing *aanat2* expression in the LL and DD conditions, as detected by WMISH and qPCR analyses, restriction of *aanat2* expression to the dark phase was evident by WMISH, but not by qPCR (Figs 1b and 2e). This is most likely due to transcripts for *aanat2* (which are restricted to the small pineal gland at 2 dpf) being heavily diluted when isolated from whole larvae, resulting in the qPCR method having very low sensitivity for *aanat2*, precluding direct comparison with the more sensitive WMISH analysis. This was evidenced by very high Ct values for *aanat2*, compared to those for the clock genes *per2a*, *cry1a*, *per1b* and *per3*. Of note, previous studies that have measured the rhythmicity of pineal gland *aanat2* expression utilize WMISH detection, rather than qPCR^31,36–40^.

The WMISH and qPCR results collectively showed that two full LD or DL cycles were sufficient to observe a diel expression pattern of the light-inducible clock genes, *per2a* and *cry1a*, and the downstream target of Per2a, *aanat2*. This confirms that the different light conditions used in this study affected the circadian rhythm at the molecular level in 2 dpf larval zebrafish.

### Light affects the survival of larval zebrafish to bacterial infection

**Figure 3:**
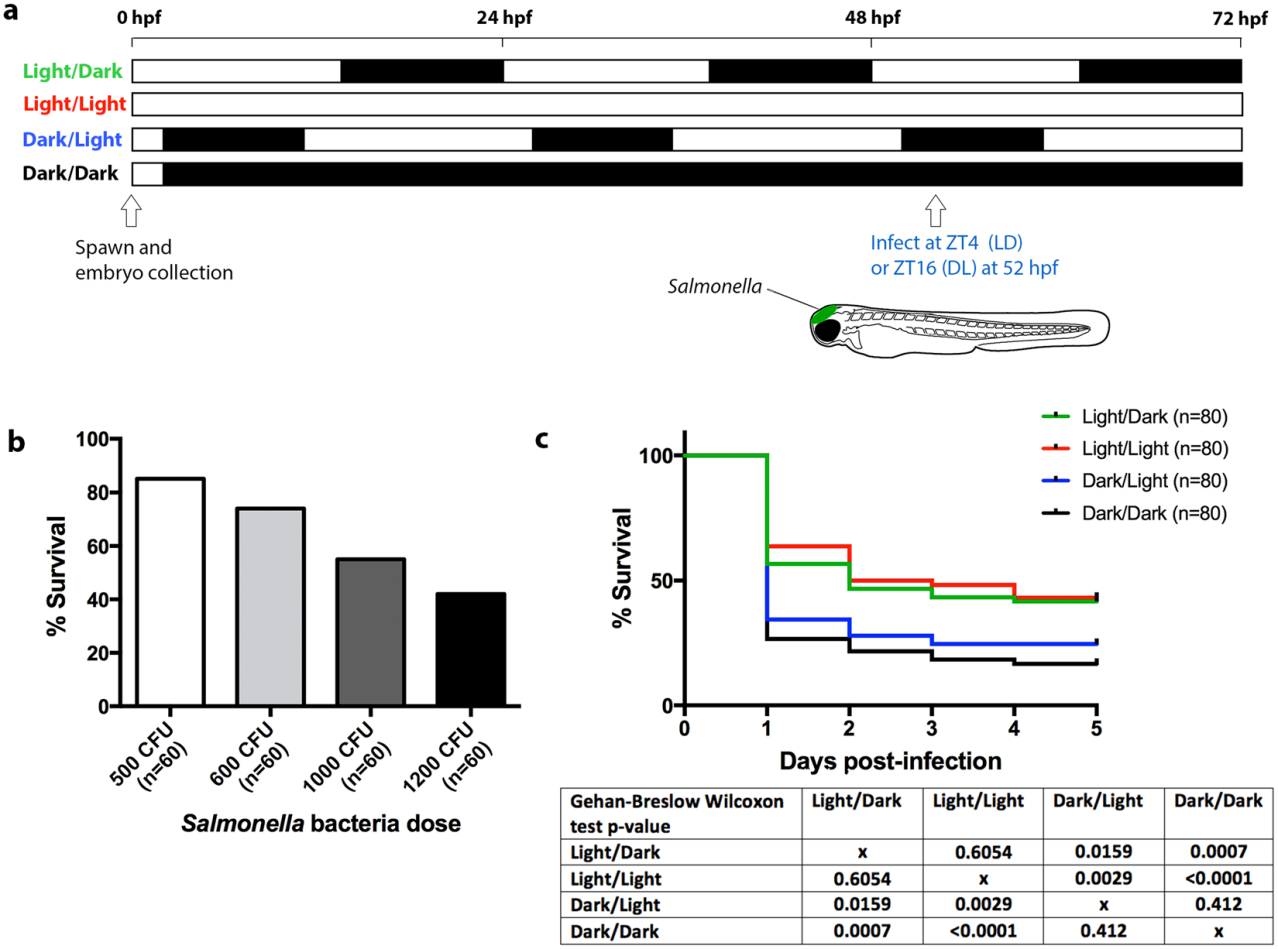
The survival of zebrafish larvae infected with *Salmonella* is higher under light exposure. (**a**) Schematic outlining the light conditions and timing of infection. (**b**) Survival outcome (% survival) of larvae infected with different doses of *Salmonella* under light/dark conditions. **(c)** Kaplan-Meier survival graph showing the % survival of infected larvae from the light/dark, constant light, dark/light and constant darkness groups from 1 to 5 days post-infection. ZT, zeitgeber time where ZT0 represents start of the light phase; hpf, hours post-fertilization.

To investigate whether light influences the innate immune response to acute infection in larval zebrafish, larvae raised under the different light conditions were infected with *Salmonella* that was delivered into the hindbrain ventricle at 52 hpf, coinciding with ZT4 under LD and ZT16 under DL (Fig. 3a). This is a well-established site for infection studies in larval zebrafish^23,24,41,42^. The infection dose of 1000 CFU was considered optimal as it resulted in ~50% survival under LD conditions (Fig. 3b). This enabled observation of both lower and higher survival rates under the different light settings. Survival was monitored for five days post-infection. This analysis revealed that the majority of mortality occurred within the first two days post-infection in all groups (Fig. 3c), consistent with previous studies using the larval zebrafish hindbrain infection model^41^. Larvae kept under DL and DD conditions displayed a considerably lower survival rate following infection compared with the LD and LL groups (Fig. 3c). The discrepancy in survival outcome between the groups infected during the light and dark phases demonstrate that the innate immune response in larval zebrafish is light-regulated.

### Light contributes to enhanced bacterial clearance

**Figure 4:**
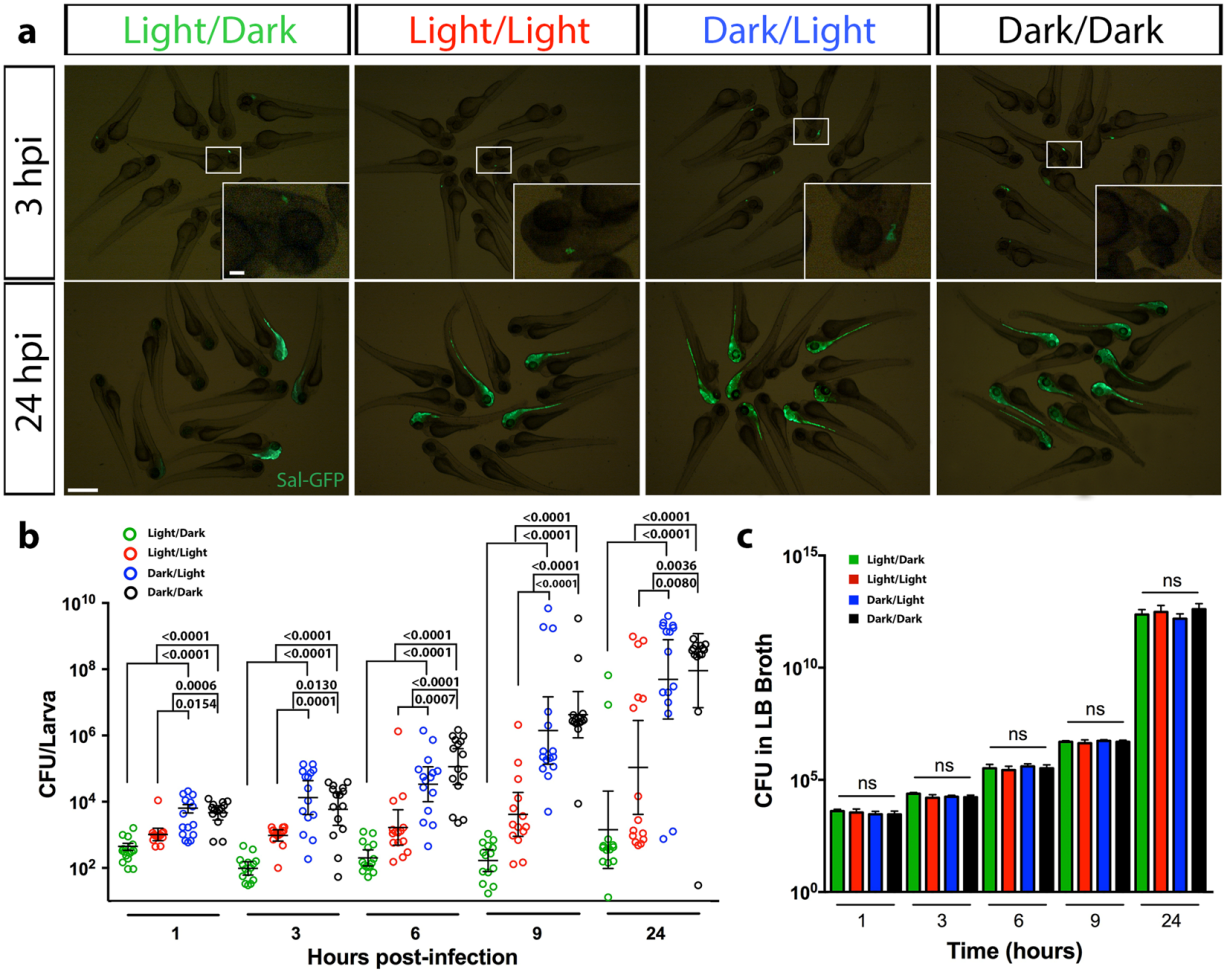
Larvae infected with *Salmonella* during light exposure demonstrate enhanced bacterial clearance. (**a**) Live imaging of larvae infected with GFP-labeled *Salmonella*, at 3 and 24 hpi under light/dark, constant light, dark/light and constant dark conditions. Inset, magnified view of boxed region. (**b**) CFU counts of GFP-labeled *Salmonella* within individual infected larvae raised under light/dark, constant light, dark/light and constant dark conditions. **(c)** CFU measurements of GFP-labeled *Salmonella* in Luria Bertani broth grown under light/dark, constant light, dark/light and constant dark conditions over a 24- hour period. Scale bar, 500 μm and 100 μm in inset; hpi, hours post-infection; data in (**c**) represent three biological replicates; data shown as mean ± s.e.m. in (**b**) and s.d. in (**c**); p-values were calculated by one-way ANOVA with Tukey’s multiple comparisons test; ns, not significant.

We next examined whether the higher survival rate following infection during light exposure was due to greater bacterial clearance. To live image the bacterial burden within infected larvae, we used a GFP-tagged *Salmonella* (Sal-GFP)^20,23^. Live fluorescence microscopy revealed diminished Sal-GFP clearance in the hindbrain in the DL- and DD-infected groups compared to the LD- and LL-infected groups (Fig. 4a). At 3 hours post-infection (hpi), if observable by fluorescence microscopy, Sal-GFP was localized to the hindbrain infection site (Fig. 4a). Larvae that eventually succumbed to death were unable to contain the infection in the hindbrain by 24 hpi, resulting in its spread, typically along the neural tube (Fig. 4a). To validate these observations, the bacterial burden within individual infected larvae was quantified at five time points post-infection: 1, 3, 6, 9 and 24 hpi (Fig. 4b). This revealed that a difference in bacterial burden between the groups was first evident at 1 hpi with the DL and DD groups having a significantly higher bacterial burden (Fig. 4b). While the bacterial burden during the first 9 hpi remained relatively constant in the LD group, there was a slight increase, although not significant, in the LL group. This was in contrast to the DL and DD groups that had a considerably higher bacterial burden (Fig. 4b). Consistent with the survival data (Fig. 3c), by 24 hpi, only a small proportion of larvae in the LD and LL groups were overwhelmed with bacteria while a substantially higher proportion of larvae in the DL and DD groups were overwhelmed (Fig. 4b). To eliminate the possibility that the bacterial clearance within infected larvae was not a reflection of the direct effect of light on bacterial growth, we measured the bacterial load in inoculated Luria Bertani broth under the same light conditions. We showed that the growth of *Salmonella* was unaffected by the light condition (Fig. 4c). These data reveal that the enhanced survival of larvae, when infected during the light phase, is the result of elevated clearance of bacteria as a consequence of a host-derived immune response.

### Light contributes to increased expression of pro-inflammatory cytokines during infection

**Figure 5:**
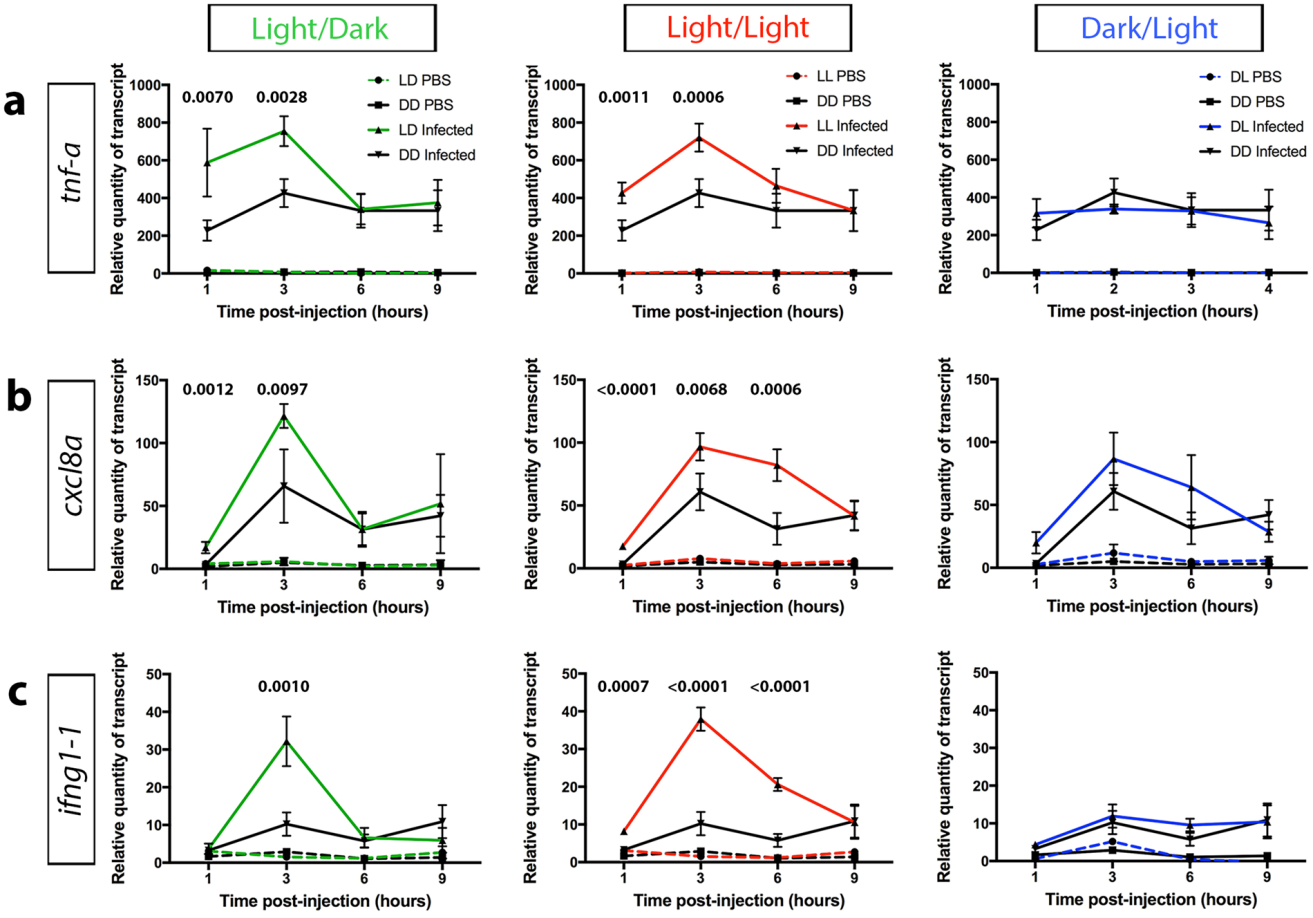
Larvae infected under light exposure demonstrate elevated expression of pro-inflammatory cytokines. (**a**–**c**) qPCR analysis of *tnf-a* **(a)** *, cxcl8a* **(b)** and *ifng1-1* **(c)**, from whole larvae raised under light/dark, constant light and dark/light conditions in comparison to constant darkness, measured at 1, 3, 6 and 9 hours post-injection. Data represent three biological replicates; data shown as mean ± s.d.; p-values were calculated by one-way ANOVA with Tukey’s multiple comparisons test; LD, light/dark; LL, constant light; DL, dark/light; DD, constant darkness.

To investigate the mechanism behind the effect of light on the innate immune response to bacterial infection, the infection-induced expression of pro-inflammatory cytokines was assessed under the different light conditions. The production and release of pro-inflammatory cytokines by innate immune cells is a critical response to infection^43,44^. Major pro-inflammatory cytokines, such as Tnf-α, IL-8 and IFN-γ, have been demonstrated to play a crucial role in the inflammatory response to infection^43,44^. Expression analysis of the zebrafish orthologues of these genes, *tnf-a* (Fig. 5a)*, cxcl8a* (Fig. 5b) and *ifng1-1* (Fig. 5c) at 1, 3, 6 and 9 hpi revealed a peak at 3 hpi with an overall greater expression in the LD and LL groups, specifically at 1 and 3 hpi, in comparison to the DD group. However, there was no significant difference in the expression level of the pro-inflammatory cytokine genes between the DL- and DD-infected groups (Fig. 5a–c) or between the various light conditions in the PBS-injected (uninfected) control groups (Supplementary Fig. 2a–c). This suggests that the greater expression of pro-inflammatory cytokines in response to infection is mediated by the presence of light and supports the notion that light promotes the innate immune response to bacterial infection.

### Light contributes to the enhanced recruitment of innate immune cells during bacterial infection

Pro-inflammatory cytokines play a significant role in the recruitment of innate immune cells to the site of infection^43,44^. We next examined whether the increased production of pro-inflammatory cytokines in response to infection under light had any impact on the recruitment of neutrophils and macrophages.

**Figure 6:**
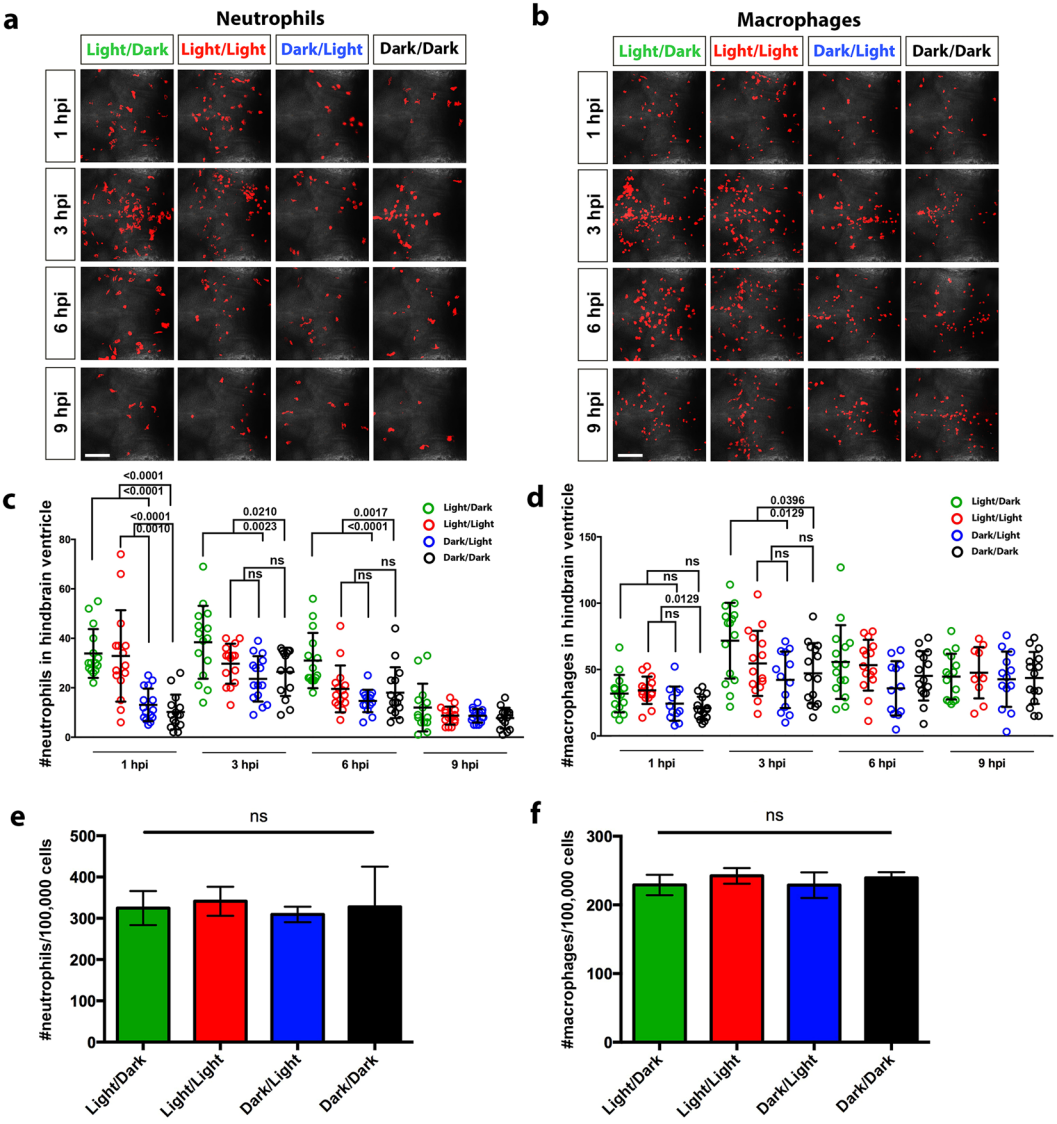
Larvae infected under light exposure demonstrate greater recruitment of neutrophils and macrophages to the hindbrain ventricle. (**a**) Immunofluorescence detection of neutrophils at 1, 3, 6 and 9 hpi within the hindbrain region of infected *Tg(lyz:DsRED2)* ^*nz50*^ larvae raised under light/dark, constant light, dark/light and constant dark conditions. (**b**) Immunofluorescence detection of macrophages at 1, 3, 6 and 9 hpi within the hindbrain region of infected *Tg(mpeg1:Gal4-FF)* ^*gl25*^ *;Tg(UAS-E1b:nfsB.mCherry)* ^*c264*^ larvae raised under light/dark, constant light, dark/light and constant dark conditions. (**c**) Quantification of neutrophils in the hindbrain region of individual infected *Tg(lyz:DsRED2)* ^*nz50*^ larvae, as detected in (**a**). (**d**) Quantification of macrophages in the hindbrain region of individual *Tg(mpeg1:Gal4-FF)* ^*gl25*^ *;Tg(UAS-E1b:nfsB.mCherry)* ^*c264*^ larvae, as detected in (**b**). (**e,f**) Flow cytometry quantification of neutrophils and macrophages from *Tg(lyz:DsRED2)* ^*nz50*^ and *Tg(mpeg1:Gal4-FF)* ^*gl25*^;*Tg(UAS-E1b*:*nfsB*.*mCherry)* ^*c264*^ larvae, respectively, raised under light/dark, constant light, dark/light and constant dark conditions. Data represent three biological replicates. Scale bar, 50 μm; data shown as mean ± s.d.; hpi, hours post-infection; p-values were calculated by one-way ANOVA with Tukey’s multiple comparisons test; ns, not significant.

This was achieved by quantifying the numbers of neutrophils and macrophages in the hindbrain of infected *Tg(lyz:DsRED2)* ^*nz50*^ and *Tg(mpeg1:Gal4-FF)* ^*gl25*^ *;Tg(UAS-E1b:nfsB.mCherry)* ^*c264*^ larvae, respectively, at 1, 3, 6 and 9 hpi under the different light conditions (Fig. 6a,b). This analysis revealed that the number of neutrophils and macrophages peaked around 3 hpi for all light conditions (Fig. 6c,d). The number of neutrophils in the hindbrain was substantially higher in the LD and LL groups in comparison to the DL and DD groups at 1 hpi but this difference persisted only for the LD group at 3 and 6 hpi (Fig. 6c). By 9 hpi, the number of neutrophils in all four groups diminished to a similar level and this was comparable to the numbers observed in the infected DD group at 1 hpi (Fig. 6c). In contrast, less variation in the number of macrophages was observed between the four groups (Fig. 6d). Only a slight increase in macrophage numbers was detected for the LD group at 3 hpi compared to the DL and DD groups whereas this difference was only seen between the LL and DD groups at 1 hpi (Fig. 6d). No significant difference was observed between the DL and DD treatments for both neutrophils and macrophages at all the examined time points (Fig. 6c,d). To assess whether this variation was the result of a direct effect of light on the development of these innate immune cells, we investigated whole-larvae numbers of neutrophils and macrophages in 2 dpf *Tg(lyz:DsRED2)* ^*nz50*^ and *Tg(mpeg1:Gal4-FF)* ^*gl25*^ *;Tg(UAS-E1b:nfsB.mCherry)* ^*c264*^ larvae, respectively, that were conditioned to the different light treatments. This analysis showed that there was no significant difference in the total numbers of neutrophils or macrophages (Fig. 6e,f).

**Figure 7:**
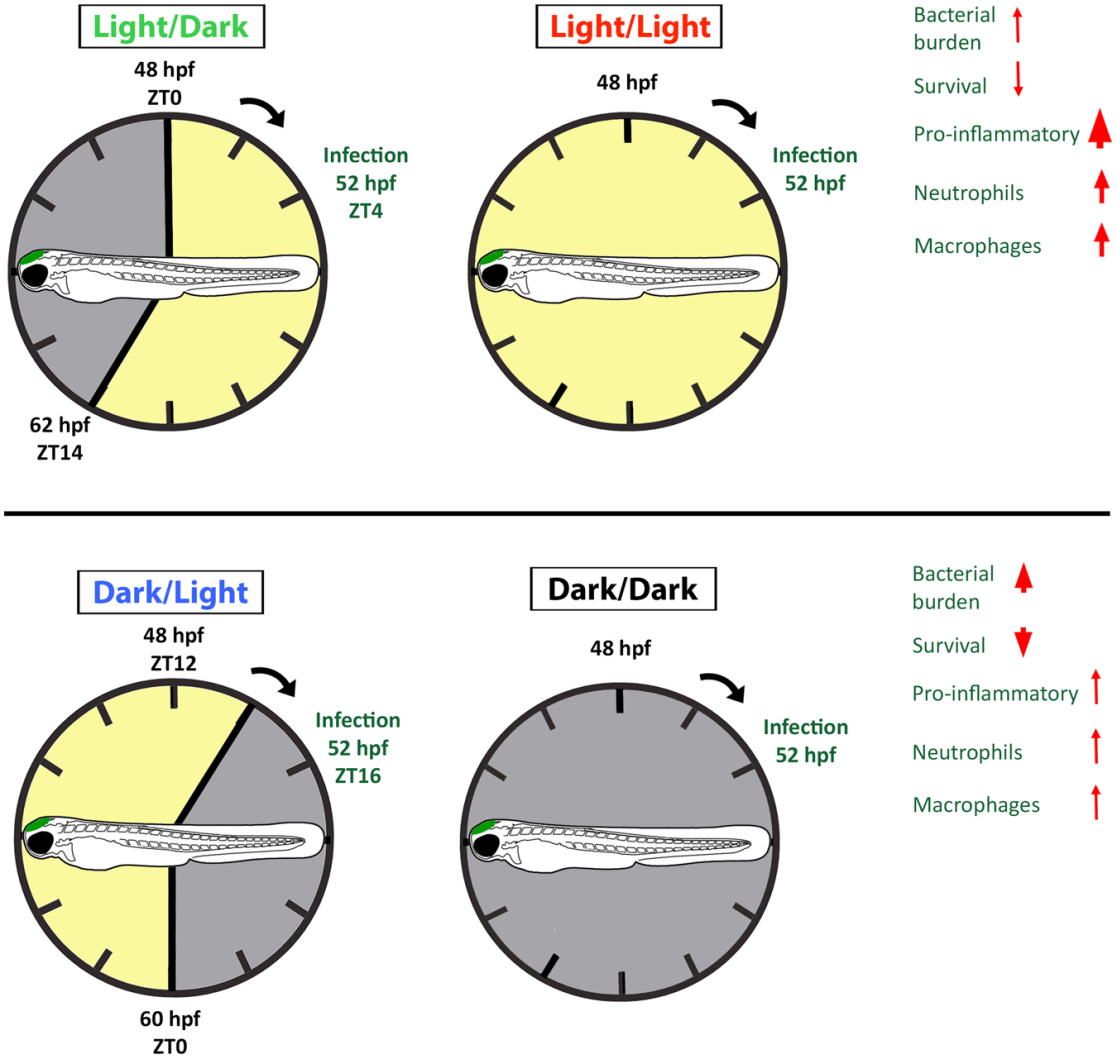
Summary of the innate immune response to bacterial infection in larval zebrafish under different light conditions. The innate immune response can be divided into two states with enhanced bacterial clearance and survival when infection occurs under light exposure in the light/dark and constant light groups. This is, at least in part, the result of greater pro-inflammatory cytokine expression and neutrophil and macrophage recruitment to the infection site. In contrast, when infected during the dark condition (either under dark/light or constant darkness) there is reduced bacterial clearance and survival that coincides with a reduction in pro-inflammatory gene expression and leukocyte recruitment.

These results suggest that greater neutrophil and, to a lesser extent, macrophage recruitment occur following infection during the light phase. This is consistent with the expression profile of the pro-inflammatory cytokines investigated earlier and confirms that the innate immune response in larval zebrafish is augmented under light exposure (summarized in Fig. 7).

## Discussion

There is increasing evidence that light can directly regulate the immune system, although this influence of light has not been explored in detail for host-microbe interactions. To uncover the dynamic mechanisms between light and the immune system in response to infection requires an alternative animal model system with high-resolution live imaging capability. Here, we present the first study to validate the use of whole-larval zebrafish, a widely used infection model that permits live imaging within a completely intact host, for investigating the innate immune response to bacteria under different light settings. Our study demonstrates that light provides a protective role against bacterial infection by promoting the innate immune response, confirming that innate immunity in larval zebrafish is light-regulated.

The inflammatory response to infection is mediated by the release of pro-inflammatory cytokines, which are vital signaling molecules important for fighting and preventing the spread of infections^43,44^. We show that following infection under light exposure, a greater pro-inflammatory response was elicited when compared with the response under darkness, as evidenced by higher expression levels of the zebrafish genes: *tnf-a, cxcl8a* and *ifng1-1*. TNF-α attracts neutrophils, which are the principle phagocytic cells, to the site of infection by upregulating chemokines, such as IL-8, and facilitates diapedisis through the upregulation of cell adhesion molecules^45,46^. Together, TNF-α and IL-8 stimulate neutrophils to degranulate, releasing antimicrobial contents, and to produce reactive oxygen species as part of the host defense against infection^43^. Further potentiation of macrophage activity is induced by IFNγ during immune activation^43^. This helps explain the trend observed between the pro-inflammatory cytokine profile and neutrophil recruitment between the light conditions, LD and LL, and dark conditions, DL and DD. It has long been recognized that macrophages are a major source of pro-inflammatory cytokines^43,44^. Despite the minor increase in macrophage recruitment under light, the significantly greater pro-inflammatory response detected may be indicative of heightened macrophage activity.

The daily variation in optimal immune activity is most likely attributed to a combination of the endogenous circadian rhythm and the daily rhythm of environmental cues^47–50^. Based on the higher survival rate under light exposure, our study demonstrates that light is an important cue for stimulating innate immune activity in larval zebrafish. However, it is still unclear whether the light-induced effects on immunity are mediated by the circadian clock or through other pathways. Given that infection under LD and LL conditions coincided with higher expression levels of *per2a* and *cry1a*, in comparison to the time-matched levels under DL and DD conditions, it is plausible that the light-inducible clock pathway plays a role in the innate immune response. PER and CRY have been identified as clock components that regulate inflammation in mice, which support the connection between light and the innate immune response to infection^2^. The expression of mammalian *Per2, Cry1* and *Cry2* genes are driven by the BMAL1:CLOCK heterodimer^2–4^. The subsequent repression of BMAL1 and CLOCK by these light-inducible clock components forms the basis of the molecular circadian oscillator, which operates in a similar manner in zebrafish^14^. So far, studies suggest PER2 may play a role in promoting inflammation as knockout of its function provides protection from LPS-induced lethality by limiting TNF-α and IL-12 production and dampens the immune response to pathogens by attenuating toll-like receptor 9 expression^51,52^. However, the exact mechanism underlying the pro-inflammatory effect of PER2 is unknown to date. It has been suggested to occur through the repression of BMAL1 activity, which has been shown to be anti-inflammatory^48^. This hypothesis fits with the antiphasic mRNA rhythms of *Per2* and *Bmal1* in macrophages^11^. Conversely, *Cry1* and *Cry2* have been suggested to mediate an anti-inflammatory effect by inhibiting NF-κВ activation via the inhibition of cAMP-dependent protein kinase A signaling^53^. Given both *per2a* and *cry1a* expression was upregulated during infection under LD and LL conditions, it is unclear how the interplay between their antagonistic effects regulate downstream immune targets and remains a subject for future research.

Additionally, the light-dependent response of the innate immune system could be a consequence of other light-directed pathways unrelated to the molecular clock. Considerable experimental evidence supports the existence of a strong bidirectional relationship between the immune and endocrine systems^6,54,55^. Light exposure modulates the endocrine glands of the hypothalamus-pituitary-adrenal axis, leading to daily changes in the level of endocrine hormones^6^. Melatonin, which is produced during the dark phase, has been described as an immunological buffer due to its stimulatory role under immunosuppressive conditions and anti-inflammatory role during immune challenges, such as acute inflammation^56^. It can reduce inflammation by blocking the downstream production of pro-inflammatory cytokines via NF-κB and reactive oxygen species due to its antioxidant properties^57^. Furthermore, melatonin limits leukocyte recruitment by reducing their adhesion and migration along the endothelium^58,59^, which possibly explains the dampened innate immune response that was observed during the dark phase in our study. Conversely, production of glucocorticoids, such as cortisol, and serotonin increases following light stimulus^6^. Like melatonin, the glucocortioids are known to have both pro-inflammatory and anti-inflammatory activities^54,60^. In the context of acute inflammation, glucocorticoids enhance the production of pro-inflammatory cytokines by upregulating the expression of inflammasome components in macrophages that are required for producing mature IL-1β as well as promoting the secretion of TNF-α and IL-6^61^. This pro-inflammatory response can also be augmented by serotonin, which stimulates downstream production of glucocorticoids^6^. This is in line with the elevated innate immune response in zebrafish larvae during light exposure. To address whether light regulates innate immunity via circadian clock-dependent or -independent pathways would require assessing the immune response to infection following selective knockout of light-inducible clock genes.

In conclusion, these data demonstrate that light positively regulates the innate immune response to bacterial infection in larval zebrafish. This was evidenced by enhanced pro-inflammatory cytokine production and recruitment of innate immune cells to the infection site. This study provides a platform to further understand the molecular mechanism through which light modulates the innate immune cell response during infection.

## Methods

### Animal maintenance

Adult zebrafish (*Danio* rerio) were raised under 14:10 h light/dark (LD) cycles at 28 °C according to standard husbandry protocols. The wild-type AB strain, *Tg*(*lyz:DsRED2)* ^*nz50*^ and *Tg(mpeg1:Gal4-FF)* ^*gl25*^ *;Tg(UAS-E1b:nfsB*.*mCherry)* ^*c264*^ lines were used in this study^20,21^. Research was conducted with approval from the University of Auckland Animal Ethics Committee (protocol AEC001343) and all experiments were conducted in accordance with this approval. Embryos were obtained within the first hour of lights on, placed in E3 Medium and raised at 28 °C under the following light conditions: LD cycle, constant light (LL), reversed 14:10 h light/dark (DL) cycle or constant darkness (DD). Zeitgeber time (ZT) is expressed in hours with ZT0 and ZT14 corresponding to light onset and dark onset, respectively. For all experiments, embryos were exposed to minimal dim light under the dissecting microscope during manual dechorination and phenylthiourea (PTU) treatment (0.003% in E3 medium) to inhibit pigmentation.

### Whole mount *in situ* hybridization (WMISH)

Transcripts of *per2a* and *aanat2* were detected using antisense, digoxigenin (DIG)-labeled riboprobe (Roche). The *per2a* expression vector was generated by cloning a 1,021 bp amplicon of the open reading frame from cDNA, using the primers *per2a* forward (5′-AAGACCTGGATTCCAAACCGT-3′) and *per2a* reverse (5′-CCGCCGCTAATACGACAGAA-3′), into the pGEM®-T Easy vector (Promega). To make *per2a* antisense riboprobe, the expression vector was linearized by *NotI* digestion and transcribed using SP6 RNA polymerase (Roche). Similarly, the *aanat2* expression vector, gifted by Professor Yoav Gothilf, Tel Aviv University Department of Neurobiology, was transcribed with Sp6 RNA polymerase following linearization with *NotI* digestion^31^. WMISH was carried out as previously described^62^. NBT/BCIP solution (Roche) was used for color precipitation to detect the DIG-labeled probes.

### RNA extraction and quantitative PCR (qPCR)

TRIzol (Invitrogen) reagent was used to extract total RNA from 30 larvae at 4-hour intervals starting from ZT0 (48 hpf) to ZT4 (76 hpf) under LD. The same time points were used for RNA extraction from larvae raised under LL, DL and DD conditions. cDNA was synthesized from 1 μg of RNA using iScript^TM^ cDNA Synthesis Kit (Bio-Rad). qPCR primers used in this study were designed using the Primer-BLAST Primer designing tool (NCBI) and are shown in Table 1.

**Table 1:** List of qPCR primers.

Primer name	Sequence (5′-3′)
*aanat2 forward*	TCGGTGTCTGGTGAATGTCC
*aanat2 reverse*	GTGTGCTCATAGCCTCCTGTT
*β-actin forward*	CGTGCTGTTTTCCCCTCCA
*β-actin reverse*	TCACCAACGTAGCTGTCTTTCTG
*cry1a forward*	GAGGGCATGAAGGTGTTCGAG
*cry1a reverse*	AAGCTCACAGGGCAATAGCA
*cxcl8a forward*	CTTAACCCATGGAGCAGAGG
*cxcl8a reverse*	CCAAGCCCACACTGTAAAGA
*ifng1-1 forward*	CGCTTGCAAAGGATTGGGTT
*ifng1-1 reverse*	GTTGGCTTAGAATTCAGCTGGT
*per1b forward*	TACAGGAGGATTCCCGCTCA
*per1b reverse*	GCTGCTCTGACTGCTTCTAGT
*per2a forward*	TCCGTCTCTGACTCCTCTGG
*per2a reverse*	CTTCAATGATCTCTTTCTTGTCTCC
*per3 forward*	CCGCTGTCAGAACGGAGATT
*per3 reverse*	ATTTAGAGGGCCAGTGCGAA
*tnf-a forward*	CAAGGCAATTTCACTTCCAA
*tnf-a reverse*	GGAGAGATGACCAGGACCAG

qPCR was performed with PerfeCTa® SYBR® Green SuperMix (Quanta Bioscience) using a QuantStudio™ 12 K Flex Real-Time PCR System (Thermo Fisher Scientific) under the conditions: 95 °C for 2 min with 40 cycles of 95 °C for 15 s and 60 °C for 30 s. Reactions were performed in technical quadruplicates, each with three biological replicates. Analysis was performed using the comparative Ct method with *β-actin* as the endogenous control, which exhibited constant expression levels across all samples and time points tested. The efficiency of each primer pair was derived and confirmed by [10^(1/−S)^−1] × 100, where S is the slope of the linear regression analysis based on reactions using 10-fold serially diluted cDNA^63^. Validation of primer performance was based on the correlation coefficient value (R^2^) > 0.99 and the specificity of the primers was confirmed by detection of a single peak using melting curve analysis.

### Live bacteria injections

Bacteria was prepared by inoculating *Salmonella* or Sal-GFP in 4 ml of Luria Bertani (LB) broth and cultured overnight at 28 °C followed by a 10-fold dilution subculture of equal parts LB and Dulbecco’s Modified Eagle Medium (DMEM) (Gibco). The subculture was incubated with shaking at 37 °C for 45 min before isolating the bacterial pellet by centrifugation and resuspension with filter-sterilized PBS supplemented with 0.25% phenol red to a final concentration of 1000 CFU/nl. Two dpf larvae were anesthetized in tricane and mounted in 3% methyl cellulose in E3 medium. Approximately 1000 CFU of bacteria was microinjected into the hindbrain ventricle at 52 hpf for all treatment groups, as previously described^23^. This dose was validated for each experiment by plating an injection bolus that was diluted 1:10 and 1:100 using filter-sterilized PBS (in duplicates) before and after injecting each cohort. The *Salmonella* growth analysis was performed by the addition of the same dose to 4 mL of LB.

### Measuring survival

Measurement of survival was performed as previously described^41^. Approximately 30 infected larvae were placed into 100 mm petri dishes to prevent overcrowding and monitored. Any dead larvae (judged by cardiac arrest) were removed. Larvae displaying excessive Sal-GFP dissemination (as detected by fluorescence microscopy) were transferred to a separate petri dish and any larval debris was removed to prevent contamination of the E3 medium with free bacteria and its spread to surviving larvae.

### Individual CFU counts

Individual CFU counts were performed as previously described^41^. In brief, infected larvae were washed in PBS three times to remove adherent bacteria. Each larva was homogenized, by repeated pipetting, in 100 μl filter-sterilized PBS supplemented with 1% Triton X-100. The dissociated larval suspension was diluted 1:10, 1:10^3^, 1:10^5^ and 1:10^7^ and 10 μl of each dilution was spot plated in triplicates onto LB agar plates containing 25 μg/mL kanamycin and incubated overnight at 28 °C. Bacterial burden was analyzed using 15 larvae from each time point per light condition.

### Immunofluorescence

Immunofluorescence was carried out as previously described with some modifications^64^. Larvae were incubated in proteinase k (10 μg/ml) for 30 min at room temperature for permeabilization before fixation in 4% paraformaldehyde. The following primary and secondary antibodies were used: mouse anti-mCherry (1:500/Clontech) with goat anti-mouse Alexa Fluor 568 (1:500/Invitrogen) and rabbit anti-DsRed (1:500/Clontech) with goat anti-rabbit Alexa Fluor 568 (1:500/Invitrogen).

### Confocal imaging

Fixed larvae were mounted in 1% low melting-point agarose (Invitrogen) and imaged using a Nikon D-Eclipse C1 confocal microscope. Z-stacks of the hindbrain ventricle were taken with a total of 40 sections at 3 μm step size intervals. Quantification of cells was achieved using Volocity v6.3 software (Perkin Elmer).

### Flow cytometry

Larvae were rinsed in ice-cold calcium-free Ringer’s solution for 15 min and then deyolked by repeated pipetting with a 200 μl tip. Larvae were digested in 0.25% trypsin-EDTA (Gibco) for 1 hour at 28 °C with manual dissociation every 10 min. The digestion was inhibited by the addition of 1 mM CaCl_2_ and 5% FBS. The dissociated cells were pelleted by centrifugation at 3000 rpm for 5 min at 4 °C before resuspension in ice-cold 0.9X PBS and 5% FBS. Cells were passed twice through 40 μm cell strainers (BD Falcon) before flow cytometry with a Becton Dickinson LSRII flow cytometer.

### Statistical analysis

Statistical analyses were performed using GraphPad Prism version 6 for Mac (GraphPad Software). Data are represented as mean ± standard deviation or standard error of mean. Statistical analyses were assessed using one-way analysis of variance (ANOVA) with Tukey’s or Dunnett’s multiple comparisons test. P-value < 0.05 was considered to be statistically significant. Cosinor analysis was performed to evaluate the presence of a 24-hour rhythm using Chronos-Fit 1.06^65^.

### Data availability

The data that support the findings of this study are available from the corresponding author upon reasonable request.

## Electronic supplementary material

Supplementary information
