# Supplementary material for: The innate immune cell response to bacterial infection in larval zebrafish is light-regulated
Source: Sci Rep. 2017 Oct 4;7:12657. doi: 10.1038/s41598-017-12842-1 (PMC5627239; doi:10.1038/s41598-017-12842-1)
Supplement: Supplementary file 1 — Supplementary information [file 41598_2017_12842_MOESM1_ESM.pdf]

SUPPLEMENTARY INFORMATION

The innate immune cell response to bacterial infection in larval zebrafish is light-regulated.

Lucia Y. Du[1], Hannah Darroch[1], Pramuk Keerthisinghe[1], Elina Ashimbayeva[1], Jonathan W. Astin[1], Kathryn E. Crosier[1], Philip S. Crosier[1], Guy Warman[2], James Cheeseman[2], Christopher J. Hall[1,*].

[1] Department of Molecular Medicine and Pathology, Faculty of Medical and Health Sciences, University of Auckland, Auckland, New Zealand.

[2] Department of Anaesthesiology, Faculty of Medical and Health Sciences, University of Auckland, Auckland, New Zealand.

[*]Corresponding author:

Christopher J. Hall

E-mail: c.hall@auckland.ac.nz

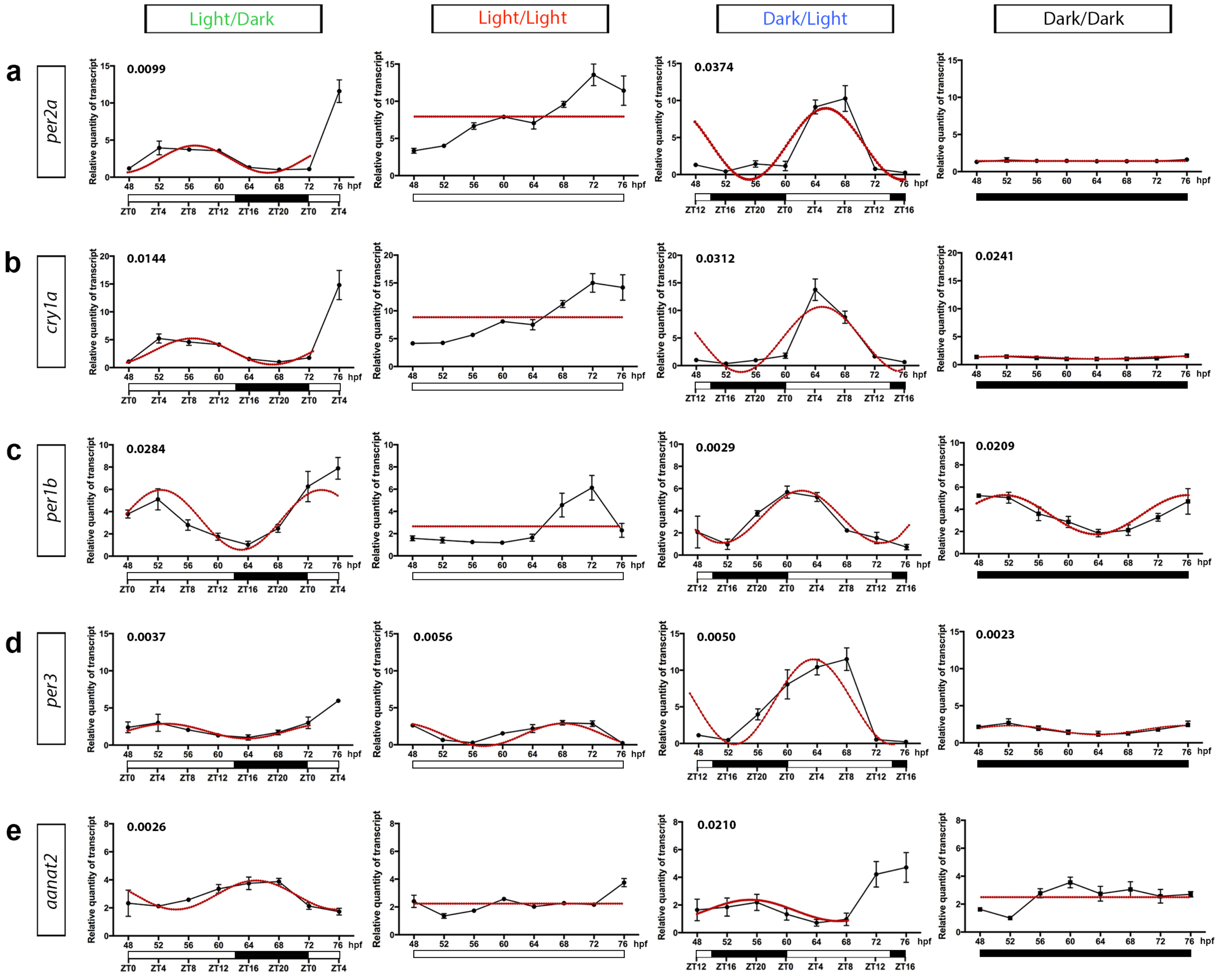

Light/Dark
Light/Light
Dark/Light
Dark/Dark
a
per2a
0.0099
0.0374
b
cry1a
0.0144
0.0312
0.0241
c
per1b
0.0284
0.0029
0.0209
d
per3
0.0037
0.0056
0.0050
0.0023
e
aanat2
0.0026
0.0210
Relative quantity of transcript
48
52
56
60
64
68
72
76
hpf
ZT0
ZT4
ZT8
ZT12
ZT16
ZT20

Supplementary Fig 1. Cosinor analysis of expression levels of clock genes and *aanat2* under different light conditions

(a-e) qPCR expression analysis of *per2a, cry1a, per1b*, *per3* and *aanat2*, respectively, from whole larvae under light/dark, constant light, dark/light and constant dark conditions from 48 hpf to 76 hpf. Red lines represent a cosinor model fitted to the log-transformed data, shown as black lines; data represents three biological replicates; data shown as mean ± s.d.; hpf, hours post-fertilization; ZT, zeitgeber time where ZT0 represents start of the light phase; *p* values were calculated by cosinor analysis.

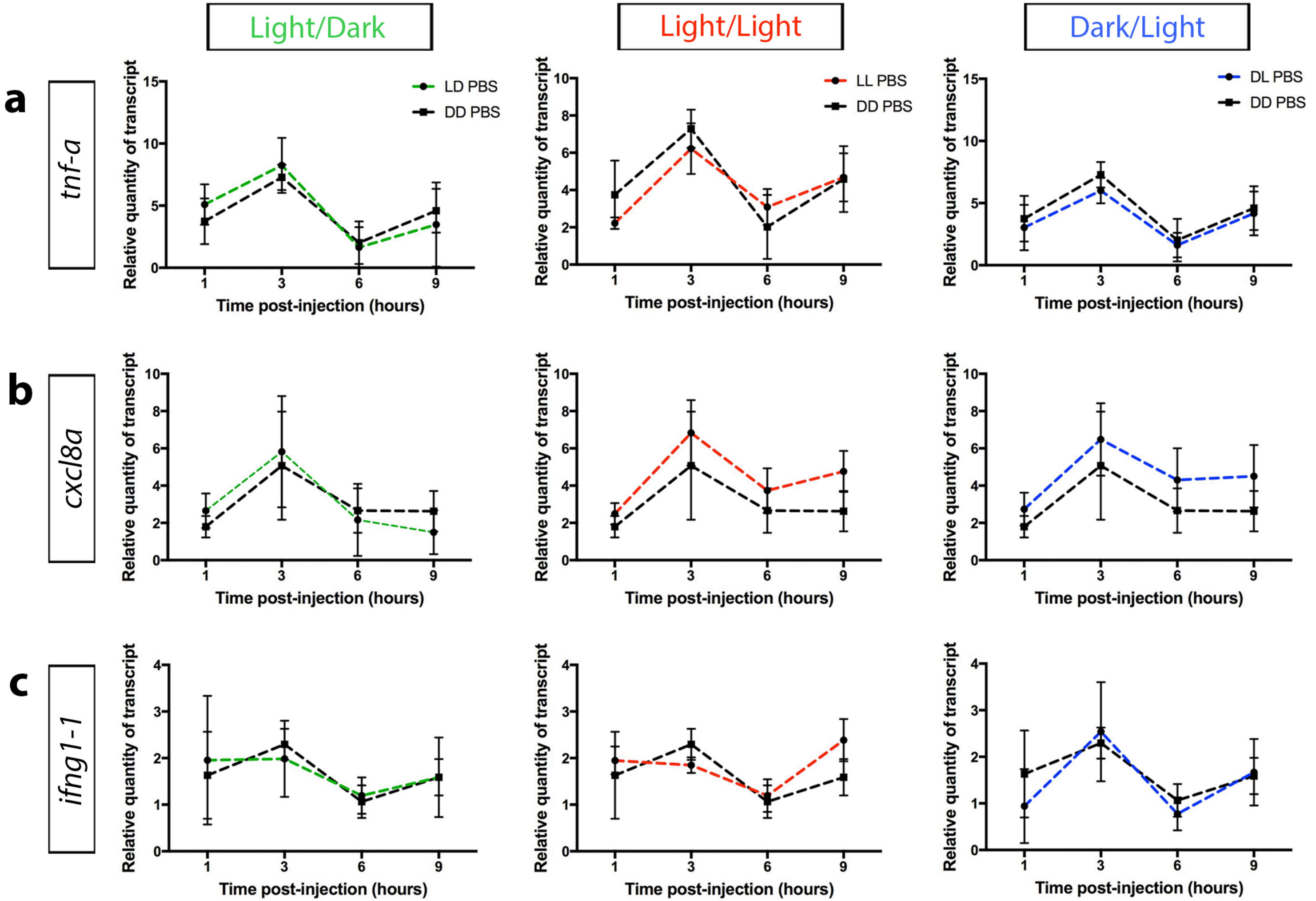
Light/Dark
Light/Light
Dark/Light
a
tnf-a
b
cxcl8a
c
ifng1-1
LD PBS
DD PBS
LL PBS
DL PBS
Relative quantity of transcript
Time post-injection (hours)

Supplementary Fig 2. Expression profiles of pro-inflammatory cytokine genes following PBS injections in larvae under different light conditions. **(a-c)** qPCR analysis of *tnf-a* **(a)***, cxcl8a* **(b)** and *ifng1-1* **(c)**, from whole PBS-injected larvae raised under light/dark, constant light and dark/light conditions in comparison to constant darkness, measured at 1, 3, 6 and 9 hours post-injection. Data represent three biological replicates; data shown as mean ± s.d.; one-way ANOVA with Tukey's multiple comparisons test revealed no significant difference; LD, light/dark; LL, constant light; DL, dark/light; DD, constant darkness.
